# Effects of an 8-week multimodal exercise program on ground reaction forces and plantar pressure during walking in boys with autism spectrum disorder

**DOI:** 10.1186/s13063-023-07158-7

**Published:** 2023-03-08

**Authors:** Mahrokh Dehghani, Amir Ali Jafarnezhadgero, Mohamad Abdollahpour Darvishani, Shirin Aali, Urs Granacher

**Affiliations:** 1 Department of Sport Managements and Biomechanics, Faculty of Educational Science and Psychology, University of Mohaghegh Ardabili, Ardabil, Iran; 2 Sport Science Department, Farhangian University, Tehran, Iran; 3 Department of Sport and Sport Science, Exercise and Human Movement Science, University of Freiburg, Freiburg, Germany

**Keywords:** Autism, Exercise, Gait analysis, Children

## Abstract

**Background:**

Autism spectrum disorder is a developmental disability with first signs appearing in children aged 3 years and younger. Given that autism spectrum disorder is accompanied by a broad range of symptoms such as impaired sensory, neurological, and neuromotor functions, it appears plausible to argue that an intervention program focusing on multimodal exercise rather than single-mode exercise might be more effective in treating this wide variety of symptoms.

**Objective:**

The aim of this study was to evaluate the effects of a multimodal exercise program entitled Sports, Play, and Active Recreation for Kids on variables of ground reaction forces and plantar pressure during walking in boys with autism spectrum disorder.

**Methods:**

Twenty-four autism spectrum disorder boys aged 7–11 years were recruited and randomly allocated into an intervention or a waiting control group. Sports, Play, and Active Recreation for Kids was conducted over a period of 8 weeks with three weekly sessions. This training protocol includes aerobic dance and jump rope exercises as well as running games. Pre- and post-training, ground reaction forces and plantar pressure variables were recorded while walking at a constant walking speed of 0.9 m/s using a foot scan embedded in a 15-m walkway.

**Results:**

Significant group-by-time interactions were found for the first peak of vertical ground reaction force, loading rate, and peak pressure at the medial heel region (all *p* = 0.001–0.49, *d* = 0.89–1.40). Post-hoc analyses showed significant pre-post decreases for the first peak of vertical ground reaction force (*p* = 0.001, *d* = 1.27), loading rate (*p* = 0.009, *d* = 1.11), and peak pressure at the medial heel region (*p* = 0.021, *d* = 1.01).

**Conclusions:**

Our results suggest that a joyful and multimodal exercise program has positive effects on kinetic walking characteristics of autism spectrum disorder boys. Accordingly, we recommend to implement this type of exercise in prepubertal autism spectrum disorder boys to improve gait kinetics.

**Trial registration:**

Iranian Registry of Clinical Trials IRCT20170806035517N4. Registered on November 8, 2021. This study was approved by the Ethical Committee of the University of Mohaghegh Ardabili, Ardabil, Iran (IR.UMA.REC.1400.019). The study was conducted in accordance with the latest version of the Declaration of Helsinki.

## Introduction

Autism spectrum disorder (ASD) is a developmental disability with first signs appearing in children aged 3 years and younger [1]. Autism spectrum disorder is a neurodevelopmental disorder estimated to affect 1 in 59 children in the USA [2]. Prevalence rates appear to be higher in boys compared with girls [3]. In fact, three out of four ASD children are boys [3]. ASD is characterized by a variety of physiological and/or behavioral symptoms including impaired sensory, neurological, and neuromotor functions resulting in muscle rigidity, akinesia, and bradykinesia [4].

While the majority of research has focused on social and neurological characteristics associated with ASD [5], motor deficits are also prominent but have received little attention in research so far. Examples of typical motor deficits observed in ASD individuals include altered walking patterns and reduced gait speed (~ 20%), particularly under more challenging conditions [6, 7]. Abnormal walking patterns may cause pain, fatigue, and increased joint stress that may lead to a decline in quality of life [8, 9]. In ASD children, there is evidence for decreased pressure under each region of the foot (especially in the hindfoot) by ~ 25% along with lower walking speed [10, 11]. Children with ASD demonstrated higher maximum braking forces (~ 30%) and lower relative times to reach the maximum braking force by ~ 22% [12]. In addition, ASD children compared with healthy controls showed a lower second peak of the vertical ground reaction force (~ 5%) during the terminal stance phase of walking [12]. These prominent differences indicate that ASD children have difficulties in supporting their body mass during the terminal stance phase which could negatively affect gait stability [13]. Taken together, ASD-related symptoms and side effects often result in low physical activity behavior in ASD children ultimately leading to adverse health effects [14]. Consequently, intervention programs should be designed and implemented with the goal to reduce ASD symptoms [15] and comorbidities [16].

Today, systematic reviews [17] and meta-analyses [18] have strengthened the available evidence on the positive effects of physical exercise on both, ASD-related symptoms (e.g., impaired neuromotor function) and comorbidities (e.g., obesity). Of note, the regular performance of exercise in the form of a graded treadmill training protocol was particularly effective in improving ASD symptoms such as walking speed with little effect on the overall health (e.g., musculoskeletal fitness) of the ASD individual [14]. Moreover, Pan [19] reported that aquatic exercise induced improvements in static balance (e.g., the center of pressure displacements during quiet standing) [20] and muscle strength (e.g., hand grip strength) in ASD children.

Given that ASD is accompanied by a broad range of symptoms such as impaired sensory, neurological, and neuromotor functions, it appears plausible to argue that an intervention program focusing on multimodal exercise rather than single-mode exercise might be more effective in treating this wide variety of symptoms. Previously, the Sports, Play, and Active Recreation for Kids (SPARK) multimodal exercise program has been introduced to promote physical activity of children aged 5–12 years [20]. There is evidence that 12 weeks of SPARK improved the gait pattern (e.g., increased step length) and gait speed in mentally retarded boys [21]. Therefore, it seems that SPARK could be a good candidate to be implemented as a treatment form in ASD children. To the best of our knowledge, there is no study available that examined the effects of a multimodal exercise program such as SPARK on walking kinetics in children with ASD. Therefore, the purpose of this study was to investigate the effects of an 8-week multimodal exercise program (SPARK) on variables of ground reaction forces and plantar pressure during walking in prepubertal boys with ASD. With reference to the relevant literature [20], we hypothesized that children with ASD who regularly participate in the SPARK program reduce their peak ground reaction force amplitudes, loading rates, and peak pressure variables during walking to a larger extent compared with ASD children enrolled in a waiting control group.

## Methods

### Participants

**Fig. 1:**
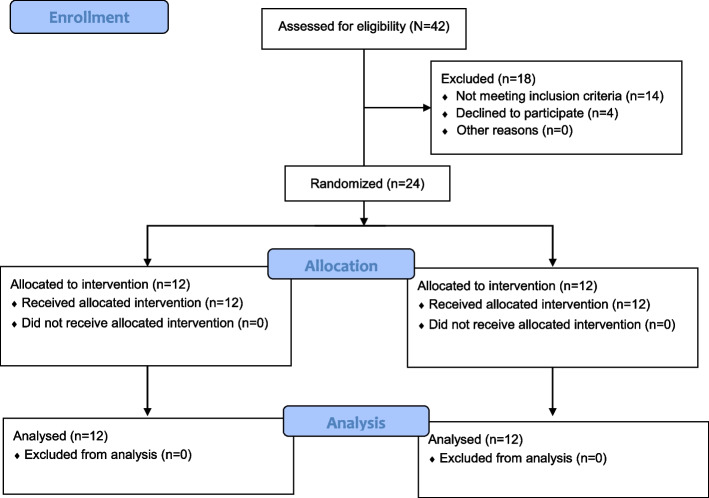
CONSORT flow diagram of the present study

We utilized the freeware tool GPower (http://www.gpower.hhu.de/) to calculate a one-sided a priori power analysis. The a priori power analysis was calculated using the *F*-test family (i.e., ANOVA repeated measures within-between interaction), and a related study that examined the effects of training on horizontal ground reaction force in ASD Children [22]. The included program variables were an assumed type I error of 0.05, a type II error rate of 0.20 (80% statistical power), and an effect size of 0.70 for horizontal ground reaction force taken from the reference study [22]. The analysis revealed that at least 12 participants would be needed per group to achieve medium- to large-sized interaction effects for the parameter horizontal ground reaction force. Accordingly, a total of twenty-four prepubertal ASD boys aged 7–11 years were recruited from a group of children who participated in an adapted physical activity program that was delivered in a local community center. The scores of the “Gilliam Autism Rating Scale-2” [23] for the participating ASD children were between 62 and 123 which shows that the participating children were diagnosed as ASD children. The enrolled boys were age-matched and randomly allocated to an experimental (*n* = 12) or a waiting control group (*n* = 12) (Fig. 1). Participants’ anthropometric and demographic data are presented in Table 1. The following information was gathered from all participating children: date of birth, intensity, and date of autism anamnesis. Children with Asperger’s or pervasive developmental disorder were excluded from this study. A medical doctor examined all participants prior to the start of the study and excluded those with neuromotor or orthopedic disorders or medication that could affect the central nervous system. None of the participants reported any secondary neurological or orthopedic conditions including lower limb injuries during the 12 months prior to data collection. ASD disorder status and the presence or absence of learning disabilities were assessed using an Iranian translation of the Social Communication Questionnaire [24, 25] and the Persian translation of the Autism Diagnostic Interview [26, 27] by the medical doctor. We obtained children’s oral consent and parents’ or legal representatives’ written consent before the start of the study. The block randomization method (block size = 4) was used to allocate study participants into the experimental groups [28]. A naïve examiner realized the block randomization process. During the randomization procedure, a set of sealed, opaque envelopes was used to ensure the concealment of the allocation. Each envelope contained a card stipulating to which group the participant would be allocated to. Of note, participants were blinded to the group allocation. One examiner determined whether a participant was eligible for inclusion, while the other carried out gait analyses of the eligible participants. Both examiners were unaware of the group allocation. Another naïve examiner (i.e., physiotherapist with 10 years of professional experience) controlled the allocation of each participant and was responsible for delivering the treatment to both groups. This study was approved by the Ethical Committee of the University of Mohaghegh Ardabili, Ardabil, Iran (IR.UMA.REC.1400.019) and registered at the Iranian Registry of Clinical Trials (IRCT20170806035517N4). The study was conducted in accordance with the latest version of the Declaration of Helsinki. The data were collected at sport biomechanics laboratory of University of Mohaghegh Ardabili, Ardabil, Iran.

**Table 1:** Group-specific baseline characteristics of the study participants

	Intervention (*n* = 12)	Waiting control (*n* = 12)	Significance level
Age (years)	9.2 ± 0.6	9.4 ± 0.5	0.904
Body mass (kg)	36.70 ± 2.47	36.70 ± 3.38	1.000
Body height (cm)	128.45 ± 4.84	130.00 ± 4.81	0.443
BMI (kg/m^2^)	22.29 ± 1.85	21.80 ± 2.61	0.599

Values are means ± standard deviations

*n* number of participants, *BMI* body mass index, *NA* not applicable

### Test procedures

During pre- and post-tests, children were instructed to walk barefoot along a 15-m walkway at a constant speed of ⁓ 0.9 m/s. In this study, walking speed was monitored and controlled using two sets of infrared photocells (Swift Performance Equipment, New South Wales, Australia). A plantar pressure plate (RsScan International, Belgium, 0.5 m × 0.5× 0.02 m, 4363 sensors) was embedded in the middle of the walkway. Formal data collection started after six familiarization trials. Thereafter, three test trials were recorded. The starting position was adjusted for each participant to make it more likely that the pressure plate was hit and that two consecutive footprints were recorded during one test trial. If the participant did not hit the pressure plate or lost his balance during the walking trials, the trial was repeated. Prior to the walking tests, a standing test was performed on the pressure-sensitive plate to record body mass together with foot length and to calibrate the system. Thereafter, the three walking test trials were performed. For data analysis, the foot was automatically divided into the following ten anatomical areas by the customized software (Footscan1 software 9 Gait 2nd Generation, Rs Scan International): medial heel (HM), lateral heel (HL), midfoot, metatarsal first to fifth (M1-5), and the hallux (T1) and other toes (T2–5). The mean of three trials was used for statistical analyses.

### Data analysis

The following dependent variables were extracted from GRF data recorded through the plantar pressure plate [29]: peak vertical GRF, their time to peak, vertical loading rate, and peak pressure (N/cm^2^) of 10 distinct regions of the foot. Of note, vertical GRF shows a bimodal curve during walking which is indicated through two peaks including the first peak during heel contact (Fz_HC_) and the second peak during the push-off phase (Fz_PO_). There is also a downfall between the two peaks (Fz_MS_) during mid-stance. To calculate the vertical loading rate during walking, the slope of the connecting line was calculated from the moment of the heel contact to the initial peak of the curve of the vertical GRF [29]. A cutoff frequency equal to 20 Hz was used to filter GRF data during walking. Ground reaction force amplitudes were normalized to body mass (BM) and reported as %BM. The gait cycle was divided into the loading (0–20%), mid-stance (20–47%), and push-off (4770%) phases. The maximum pressure (peak force/area of related region) was calculated for all ten anatomical zones before and after the 8 weeks training program. Walking data were recorded during pre and post-tests.

Intraclass correlation coefficients (ICC) were calculated for all analyzed variables using pre- and post-data from the control group. In accordance with Koo and Li, test-retest reliability in the form of ICCs was computed using two-way mixed models [30]. ICC values less than 0.5 are indicative of poor reliability, values between 0.5 and 0.75 indicate moderate reliability, values between 0.75 and 0.9 indicate good reliability, and values greater than 0.90 indicate excellent reliability [30]. Table 2 provides ICC values for all assessed parameters. Of note, sufficient test-retest reliability is important as a marker of the measurement error (noise). This is particularly important in intervention studies with pre-post design.

**Table 2:** Intraclass correlation coefficients (ICC) for all analyzed variables using pre- and post-data from the waiting control group

Variable	Component	ICCs
Vertical ground reaction force	Fz_HC_	0.90
	Fz_MS_	0.91
	Fz_PO_	0.88
Time to peak force	Fz_HC_	0.92
	Fz_MS_	0.90
	Fz_PO_	0.88
Loading rate	Vertical	0.91
Walking stance time (ms)	Walking stance time (ms)	0.86
Peak pressure	Toe 1	0.84
	Toes 2–5	0.79
	Metatarsal 1	0.85
	Metatarsal 2	0.77
	Metatarsal 3	0.77
	Metatarsal 4	0.74
	Metatarsal 5	0.75
	Midfoot	0.79
	Medial heel	0.81
	Lateral heel	0.74

*Fz*_*HC*_ peak vertical ground reaction force at heel contact, *Fz*_*MS*_ vertical ground reaction force during mid-stance, *Fz*_*PO*_ peak vertical ground reaction force during the push-off phase

### The multimodal exercise program

Training was conducted over a period of 8 weeks with three weekly sessions, each lasting 45 min. The exercise program resembled the SPARK intervention program that involves exercise and free play. Each exercise session lasted 45 min and was divided into four parts. During the first 10 min, a warm-up program was conducted consisting of stretching, walking, and jogging exercises. Thereafter, children played and exercised for 25 min to specifically promote their fundamental movement skills (e.g., jumping) through the SPARK intervention. During the main part of the SPARK session, health- and skill-related physical fitness were promoted through exercise and free play [31]. Health-related fitness exercises comprised 13 activities that included aerobic dance, running games, and jump rope exercises [20]. Accordingly, the main focus was to develop cardiovascular endurance. This was realized through the systematic programming of intensity, duration, and complexity of the respective activities [20]. Sports, Play, and Active Recreation for Kids tries to promote skill-related fitness by focusing on different sports such as soccer, basketball, and Frisbee [31]. Finally, a 10-min cool-down program consisting of dynamic stretching was realized. Over the course of the intervention period, the waiting control group performed their regular physical activity program including walking and free play. All sessions of the intervention and the waiting control group were supervised by physiotherapist who had at least 10 years of professional experience in delivering physical education to children with developmental disorders (i.e., ASD children). Overall, the intervention program included 24 SPARK sessions.

### Statistical analyses

The normal distribution of data was assessed and confirmed using the Shapiro-Wilk test. The baseline between-group differences was computed using the independent samples *t*-test. To elucidate the effects of the intervention versus the waiting control group over time, a 2 (group: exercise vs control) × 2 (time: pretest vs posttest) analysis of variance (ANOVA) with repeated measures was computed. In case statistically significant group-by-time interaction effects were established, Bonferroni-adjusted post hoc analyses (paired sample *t*-tests) were calculated. Additionally, effect sizes were determined by converting partial eta-squared (*η*^2^_*p*_) from ANOVA output to Cohen’s *d*. Within-group effect sizes were calculated using the following equation: mean difference of pre and post-tests/pooled standard deviation. According to Cohen, *d* < 0.50 indicates small effects, 0.50 ≤ *d* < 0.80 indicates medium effects, and *d* ≥ 0.80 indicates large effects [32]. The significance level was set at *p* < 0.05. The statistical analyses were computed using SPSS (version 24, SPSS Inc., 8 Chicago, IL).

## Results

All participants received treatment as allocated. The adherence rate for SPARK and the regular physical activity program was 100% for the intervention (SPARK) and the waiting control group (regular program). No training or test-related injuries were reported over the course of the study.

There were no significant between-group baseline differences for demographic and anthropometric data (Table 1).

Table 2 shows the test-retest reliability for all analyzed variables using ICCs. ICCs ranged from 0.74 to 0.92 indicating good-to-excellent reliability.

Table 3 indicates no statistically significant between-group baseline differences for measures of ground reaction force, loading rate, and peak pressure (*p* > 0.05).

**Table 3:** Group-specific baseline data for vertical ground reaction force (% of body mass), time to peak force (ms), loading rate (N/kg/s), and peak pressure (kPa)

Variable	Component	Intervention, mean ± SD	Waiting control, mean ± SD	95% CI	*p*-value
Vertical ground reaction force	Fz_HC_	1014.34 ± 168.70	947.11 ± 221.76	− 12.44, 10.86	0.412
	Fz_MS_	736.03 ± 91.85	743.92 ± 167.90	− 20.49, 20.01	0.888
	Fz_PO_	969.86 ± 221.30	947.27 ± 197.53	− 15.51, 20.03	0.794
Time to peak forces	Fz_HC_	159.83 ± 32.04	163.19 ± 50.43	− 30.37, 23.65	0.799
	Fz_MS_	296.16 ± 50.43	283.05 ± 49.12	− 29.41, 18.72	0.526
	Fz_PO_	438.75 ± 60.94	411.66 ± 70.10	− 29.03, 55.25	0.323
Loading rate	Vertical	6.53 ± 1.51	5.89 ± 1.31	− 0.55, 1.84	0.279
Walking stance time (ms)	Walking stance time (ms)	612.19 ± 82.68	600.27 ± 68.49	− 52.36, 30.99	0.704
Peak pressure	Toe 1	88.43 ± 21.70	77.80 ± 22.29	− 8.00, 29.25	0.249
	Toe 2-5	35.87 ± 18.08	38.95 ± 19.06	− 18.81, 12.66	0.688
	Metatarsal 1	67.44 ± 14.78	63.34 ± 9.10	− 6.28, 14.50	0.423
	Metatarsal 2	98.09 ± 29.21	87.50 ± 28.63	− 13.90, 35.07	0.380
	Metatarsal 3	78.96 ± 19.76	75.63 ± 12.16	− 10.56, 17.22	0.624
	Metatarsal 4	64.90 ± 24.52	61.04 ± 14.97	− 13.34, 21.06	0.647
	Metatarsal 5	46.38 ± 15.85	52.29 ± 22.62	− 22.45, 10.26	0.466
	Midfoot	36.29 ± 12.33	41.19 ± 8.75	− 13.95, 4.15	0.274
	Medial heel	95.50 ± 28.49	78.43 ± 29.28	− 14.68, 29.60	0.162
	Lateral heel	83.70 ± 22.67	76.24 ± 29.22	− 14.75, 29.68	0.492

*SD* standard deviation, *Fz*_*HC*_ peak vertical ground reaction force during heel contact, *Fz*_*MS*_ vertical ground reaction force during mid-stance, *Fz*_*PO*_ peak vertical ground reaction force during the push-off phase

### Ground reaction forces during walking

The statistical analysis did not demonstrate any significant main effect of “time” (*p* > 0.05, *d* = 0.06–0.81) for vertical ground reaction forces during walking at a constant speed, their time-to-peak, loading rate, and walking stance time (Table 4). We observed significant group-by-time interactions for the first peak of the vertical ground reaction force, time-to-peak until the second peak of the vertical ground reaction force, and loading rate (*p* < 0.049, *d* = 0.89–0.91) (Table 4). Pair-wise analyses demonstrated that the intervention but not the control group showed significant decreases for the first peak of vertical ground reaction force (*p* = 0.001, *d* = 1.27), time-to-peak until the second peak of vertical ground reaction force (*p* = 0.012, *d* = 1.28), and loading rate (*p* = 0.009, *d* = 1.11).

**Table 4:** Group-specific mean values and standard deviations for vertical ground reaction force (% of body mass), time to peak force (ms), and loading rate (N/kg/s) during walking at constant speed in ASD boys

Variable	Component	Intervention				Waiting control				Main effect of time (*p*-value, Cohen’s *d*)	Main effect of group (*p*-value, Cohen’s *d*)	Group × time interaction (*p*-value, Cohen’s *d*)
		Pre	Post	Δ %	95% CI	Pre	Post	Δ %	95% CI			
Vertical ground reaction force	Fz_HC_	**1014.34 ± 168.70**	**811.83 ± 147.89**	− 19.96	106.71–298.29	947.11 ± 221.76	959.56±138.39	1.31	− 210.78–185.89	0.071 (0.810)	0.424 (0.346)	0.043* (0.915)
	Fz_MS_	736.03 ± 91.85	672.06 ± 162.05	− 8.69	− 71.50–199.44	743.92 ± 167.90	671.38±176.23	− 9.75	− 11.35–156.42	0.073 (0.804)	0.944 (0.000)	0.907 (0.063)
	Fz_PO_	969.86 ± 221.30	856.46 ± 219.21	− 11.69	− 97.34–324.14	947.27 ± 197.53	923.34±213.48	− 2.52	− 166.66–214.51	0.299 (0.454)	0.708 (0.168)	0.496 (0.293)
Time to peak force	Fz_HC_	159.83 ± 32.04	156.74 ± 16.21	− 1.93	− 19.94–26.11	163.19 ± 50.43	159.41±38.40	− 2.31	− 31.71–39.27	0.725 (0.155)	0.712 (0.155)	0.971 (0.000)
	Fz_MS_	296.16 ± 50.43	290.66 ± 48.68	− 1.85	− 32.97–43.97	283.05 ± 49.12	284.77±37.82	0.60	− 41.89–38.44	0.883 (0.063)	0.514 (0.286)	0.778 (0.127)
	Fz_PO_	**438.75 ± 60.94**	**373.74 ± 27.75**	− 14.81	17.63–112.37	411.66 ± 70.10	417.79±37.76	1.48	− 63.53–51.27	0.096 (0.742)	0.516 (0.278)	0.047* (0.896)
Loading rate	Vertical	**6.53 ± 1.51**	**5.18 ± 0.82**	− 20.67	0.42–2.28	5.89 ± 1.31	6.32±1.66	7.30	− 2.06–1.19	0.293 (0.459)	0.493 (0.300)	0.048* (0.896)
Walking stance time (ms)		612.19±82.68	596.86 ± 65.06	− 2.50	− 47.76–78.43	600.27 ± 68.49	599.05 ± 59.52	− 0.20	− 49.64–52.09	0.657 (0.191)	0.824 (0.090)	0.705 (0.168)

*Fz*_*HC*_ peak vertical ground reaction force at heel contact, *Fz*_*MS*_ vertical ground reaction force during mid-stance, *Fz*_*PO*_ peak vertical ground reaction force during the push-off phase, *CI* confidence interval, *d* Cohen’s *d*

^*^Significant level *P* < 0.05

### Peak pressure during walking

The statistical analysis did not demonstrate any significant main effect of “time” for peak pressure (*p* > 0.05, *d* = 0.00–0.51) during walking at a constant speed (Table 5). We observed significant group-by-time interactions for peak pressure at the medial heel region (*p* = 0.003, *d* = 1.40) (Table 5). Only the intervention group showed significant decreases from pre-to-post for peak pressure at the medial heel region (*p* = 0.021, *d* = 1.01).

**Table 5:** Group-specific mean values and standard deviations for peak pressure (kPa) during walking at constant speed in ASD boys

Variable	Intervention				Waiting control				Main effect of time (*p*-value, Cohen’s *d*)	Main effect of group (*p*-value, Cohen’s *d*)	Group × time interaction (*p*-value, Cohen’s *d*)
	Pre	Post	Δ %	95% CI	Pre	Post	Δ %	95% CI			
Toe 1	88.43 ± 21.70	69.98 ± 19.27	− 20.86	− 2.34–39.24	77.80 ± 22.29	82.62 ± 19.95	6.19	− 18.77–9.14	0.244 (0.510)	0.875 (0.063)	0.053 (0.873)
Toes 2–5	35.87 ± 18.08	33.16 ± 11.27	− 7.55	− 8.42–13.83	38.95 ± 19.06	34.70 ± 13.11	− 10.91	− 8.96–17.47	0.385 (0.375)	0.654 (0.191)	0.845 (0.090)
Metatarsal 1	67.44 ± 14.78	68.19 ± 19.59	1.11	− 18.07–16.58	63.34 ± 9.10	68.43 ± 9.66	8.03	− 15.02–4.84	0.527 (0.271)	0.580 (0.238)	0.636 (0.201)
Metatarsal 2	98.09 ± 29.21	102.90 ± 36.91	4.90	− 33.27–23.64	87.50 ± 28.63	97.67 ± 24.59	11.62	− 37.56–17.23	0.413 (0.358)	0.359 (0.397)	0.768 (0.127)
Metatarsal 3	78.96 ± 19.76	85.33 ± 25.95	8.06	− 28.92–16.18	75.63 ± 12.16	78.79 ± 18.62	4.17	− 14.59–8.28	0.416 (0.352)	0.392 (0.369)	0.782 (0.127)
Metatarsal 4	**64.90 ± 24.52**	**75.45 ± 28.24**	16.25	− 35.44–14.32	61.04 ± 14.97	51.71 ± 13.48	− 15.28	− 2.67–21.32	0.923 (0.000)	**0.031 (0.984)**	0.127 (0.674)
Metatarsal 5	46.38 ± 15.85	55.26 ± 21.35	19.14	− 24.79–7.03	52.29 ± 22.62	51.46 ± 18.51	− 1.58	− 20.46–22.12	0.512 (0.286)	0.845 (0.090)	0.430 (0.346)
Midfoot	36.29 ± 12.33	42.88 ± 16.19	18.15	− 18.15–4.98	41.19 ± 8.75	38.96 ± 11.31	− 5.41	− 4.85–9.30	0.487 (0.300)	0.904 (0.063)	0.167 (0.612)
Medial heel	**95.50 ± 28.49**	**73.01 ± 13.25**	− 23.54	4.17–40.79	78.43 ± 29.28	93.74 ± 28.82	19.52	− 29.45–12.33	0.540 (0.263)	0.839 (0.090)	**0.003 (1.400)**
Lateral heel	83.70 ± 22.67	88.19 ± 21.89	5.36	− 29.95–20.97	76.24 ± 29.22	84.80 ± 29.88	11.22	− 32.84–2.22	0.393 (0.369)	0.484 (0.300)	0.788 (0.110)

*CI* confidence interval, *d* Cohen’s *d*

## Discussion

The main findings of this study were that compared with a waiting control group, the intervention group showed (i) declines in peak vertical GRFs during heel contact, (ii) a decrease in loading rate during the loading phase of walking, and (iii) a decrease in peak pressure of the medial heel region during walking at a constant speed. Taken together, the observed findings are in accordance with our study hypotheses.

### Ground reaction forces during walking

In general, there is evidence that ASD children aged 4–12 years compared with healthy controls show higher maximal braking forces and lower relative times to reach the maximum braking force when walking at the preferred speed [12]. A greater maximal braking force in ASD children during walking may reflect a high demand in terms of weight-bearing stability and shock absorption during the first part of the stance phase [12]. Throughout the early stance phase, ASD children may encounter problems with the alignment of the lower limbs due to the rapid transfer of body mass to the limb that has just accomplished the swing phase [33]. It has previously been reported that increased loading rates and impact shocks (i.e., the first peak of the vertical ground reaction force) during walking may represent biomechanical factors associated with an increased risk of sustaining orthopedic injuries such as knee degenerative joint disease or stress fractures [34]. The SPARK exercise program induced a decrease in the first peak of vertical ground reaction force and vertical loading rates during walking in ASD boys which might contribute to lowering the injury risk in ASD children.

### Peak pressure during walking

The gait pattern of ASD children is characterized by slow walking speed and toe walking [35, 36]. More specifically, there is evidence from previous research that ASD children walk with lower pressure in the heel region accompanied by reduced walking speed [10]. Previous studies have shown that pressure distribution is affected by walking speed [10, 37, 38]. At slow walking speeds, pressure is low underneath the heel region [10, 37, 38]. Besides slow walking speed, ASD-related toe walking might also be responsible for low pressure in the heel region [35, 36]. The SPARK exercise program induced a decrease in peak pressure of the medial heel region during walking at constant speed in ASD boys. This finding implies that SPARK may have caused a shift of the plantar pressure component towards the lateral column of the foot. This again may allow greater ankle stability [39]. This is in line with another study showing that SPARK has the potential to improve the static and dynamic balance of ASD children aged 5–12 years [20]. Overall, our findings suggest that SPARK has the potential to alter the plantar pressure distribution during walking through a correction of toe walking in ASD children. Cause-effect relations have to be established in future studies.

This study has a few limitations that should be discussed. First, the number of study participants was relatively small. However, we conducted an a priori power analysis, and the findings supported our initial cohort size. Second, we did not record kinematic data as well as muscular activity in this study. These additional biomechanical analyses should be realized in future research to deduce the underlying neuromuscular mechanisms of training-induced adaptations. Another limitation of this study is that we did not apply any cardiorespiratory fitness tests. Future studies should therefore examine the effects of physical exercise on biomechanic walking characteristics and cardiorespiratory fitness in boys with autism spectrum disorder.

## Conclusions

The results of this study suggest that a multimodal exercise program (SPARK) has positive effects on the loading rate during walking at constant speed in ASD boys. Moreover, we were able to show that SPARK induced a shift of the plantar pressure component towards the lateral column of the foot which may enhance ankle stability. Accordingly, we recommend to implement SPARK because it is a safe, joyful, and effective treatment form for prepubertal ASD boys. Eight weeks of SPARK training with three weekly sessions, each lasting 45 min constitutes a sufficient exercise stimulus to improve the walking pattern of 9-year-old boys with autism spectrum disorder.

## Data Availability

Data will be available at request.

## References

[1] Karimi P, Kamali E, Mousavi SM, Karahmadi M (2017). Environmental factors influencing the risk of autism. J Res Med Sci..

[2] Zablotsky B, Black LI, Maenner MJ, Schieve LA, Blumberg SJ. Estimated prevalence of autism and other developmental disabilities following questionnaire changes in the 2014 National Health Interview Survey. Natl Health Stat Report. 2015;(87):1–20. 26632847

[3] Sigman M, Arbelle S, Dissanayake C (1995). Current research findings on childhood autism. Can J Psychiatry.

[4] Association AP, Association AP (2013). Diagnostic and statistical manual of mental disorders: DSM-5.

[5] Bhat AN, Landa RJ, Galloway JC (2011). Current perspectives on motor functioning in infants, children, and adults with autism spectrum disorders. Phys Ther.

[6] Morrison S, Armitano CN, Raffaele CT, Deutsch SI, Neumann SA, Caracci H (2018). Neuromotor and cognitive responses of adults with autism spectrum disorder compared to neurotypical adults. Exp Brain Res.

[7] Kindregan D, Gallagher L, Gormley J. Gait deviations in children with autism spectrum disorders: a review. Autism Res Treat. 2015:741480, 8 pages. 10.1155/2015/741480. 10.1155/2015/741480 PMC4398922 25922766

[8] Shetreat-Klein M, Shinnar S, Rapin I (2014). Abnormalities of joint mobility and gait in children with autism spectrum disorders. Brain Dev.

[9] Calhoun M, Longworth M, Chester VL (2011). Gait patterns in children with autism. Clin Biomech.

[10] Lim B-O, O’Sullivan D, Choi B-G, Kim M-Y (2016). Comparative gait analysis between children with autism and age-matched controls: analysis with temporal-spatial and foot pressure variables. J Phys Ther Sci.

[11] Yang C-S, Lee G-S, Choi B-K, O’Sullivan D, Kwon Y-H, Lim B-O (2012). Gait analysis in children with autism using temporal-spatial and foot pressure variables. ISBS-Conf Proc Arch.

[12] Hasan CZC, Jailani R, Tahir NM, Ilias S (2017). The analysis of three-dimensional ground reaction forces during gait in children with autism spectrum disorders. Res Dev Disabil.

[13] Hasan C, Jailani R, Tahir N, Desa H (2018). Vertical ground reaction force gait patterns during walking in children with autism spectrum disorders. Int J Eng.

[14] Srinivasan SM, Pescatello LS, Bhat AN (2014). Current perspectives on physical activity and exercise recommendations for children and adolescents with autism spectrum disorders. Phys Ther.

[15] Baghdadli A, Pry R, Michelon C, Rattaz C (2014). Impact of autism in adolescents on parental quality of life. Qual Life Res.

[16] Broder-Fingert S, Brazauskas K, Lindgren K, Iannuzzi D, Van Cleave J (2014). Prevalence of overweight and obesity in a large clinical sample of children with autism. Acad Pediatr.

[17] Lang R, Koegel LK, Ashbaugh K, Regester A, Ence W, Smith W (2010). Physical exercise and individuals with autism spectrum disorders: a systematic review. Res Autism Spectr Disord.

[18] Tan BW, Pooley JA, Speelman CP (2016). A meta-analytic review of the efficacy of physical exercise interventions on cognition in individuals with autism spectrum disorder and ADHD. J Autism Dev Disord.

[19] Pan C-Y (2011). The efficacy of an aquatic program on physical fitness and aquatic skills in children with and without autism spectrum disorders. Res Autism Spectr Disord.

[20] Najafabadi MG, Sheikh M, Hemayattalab R, Memari A-H, Aderyani MR, Hafizi S (2018). The effect of SPARK on social and motor skills of children with autism. Pediatr Neonatol.

[21] Rostamzadeh Z (2019). Effect of a spark training course on walking parameters and pulmonary capacity in educable mentally retarded boys.

[22] Gürol B (2019). Analysis of gait patterns in individuals with autism spectrum disorder after recreational therapy program at Eskisehir Technical University. Int Educ Stud.

[23] Diken IH, Diken O, Gilliam JE, Ardic A, Sweeney D (2012). Validity and reliability of Turkish version of Gilliam Autism Rating Scale-2: results of preliminary study. Int J Spec Educ.

[24] Rutter M, Bailey A, Lord C, Cianchetti C, Fancello GS (2007). SCQ: Social Communication Questionnaire: Manuale.

[25] Sasanfar R, Ghadami M (2006). Standardising and normalizing the Social Communication Questionnaire.

[26] Rutter M, Le Couteur A, Lord C (2003). Autism diagnostic interview-revised.

[27] Sasanfar R, Toloie A. Standardising and normalizing the autism diagnostic interview-revised on Iranian population. In: The Iranian special education organisation Tehran: The Iranian Special Education Organisation Publication. 2006;25(4):22–30.

[28] Lachin JM, Matts JP, Wei L (1988). Randomization in clinical trials: conclusions and recommendations. Control Clin Trials.

[29] Farahpour N, Jafarnezhad A, Damavandi M, Bakhtiari A, Allard P (2016). Gait ground reaction force characteristics of low back pain patients with pronated foot and able-bodied individuals with and without foot pronation. J Biomech.

[30] Koo TK, Li MY (2016). A guideline of selecting and reporting intraclass correlation coefficients for reliability research. J Chiropr Med.

[31] Mostafavi R, Ziaee V, Akbari H, Haji-Hosseini S (2013). The effects of spark physical education program on fundamental motor skills in 4-6 year-old children. Iran J Pediatr.

[32] Cohen J. Statistical power analysis for the behavioral sciences. New York University: Routledge; 2013.

[33] Perry J, Davids JR (1992). Gait analysis: normal and pathological function. J Pediatr Orthop.

[34] Watemberg N, Waiserberg N, Zuk L, Lerman-Sagie T (2007). Developmental coordination disorder in children with attention-deficit–hyperactivity disorder and physical therapy intervention. Dev Med Child Neurol.

[35] Uljarević M, Hedley D, Alvares GA, Varcin KJ, Whitehouse AJ (2017). Relationship between early motor milestones and severity of restricted and repetitive behaviors in children and adolescents with autism spectrum disorder. Autism Res.

[36] Valagussa G, Trentin L, Signori A, Grossi E (2018). Toe walking assessment in autism spectrum disorder subjects: a systematic review. Autism Res.

[37] Vilensky JA, Damasio AR, Maurer RG (1981). Gait disturbances in patients with autistic behavior: a preliminary study. Arch Neurol.

[38] Zhu H, Wertsch JJ, Harris GF, Alba HM (1995). Walking cadence effect on plantar pressures. Arch Phys Med Rehabil.

[39] Huang PY, Lin CF, Kuo LC, Liao JC (2011). Foot pressure and center of pressure in athletes with ankle instability during lateral shuffling and running gait. Scand J Med Sci Sports.

